# Shaping global vaccine acceptance with localized knowledge: a report from the inaugural VARN2022 conference

**DOI:** 10.1186/s12919-023-00280-z

**Published:** 2023-10-05

**Authors:** Talya Underwood, Kathryn L. Hopkins, Theresa Sommers, Cassidy Howell, Nicholas Boehman, Meredith Dockery, Ève Dubé, Baldeep K. Dhaliwal, Abdul M. Kazi, Rupali Limaye, Rubina Qasim, Holly Seale, Freddy Eric Kitutu, Robert Kanwagi, Stacey Knobler

**Affiliations:** 1 Consultant to Sabin Vaccine Institute, Northwich, UK; 2 Sabin Vaccine Institute, 2175 K Street NW, Suite 400, Washington DC, 20037 USA; 3 Quebec National Institute of Public Health, Quebec City, Canada; 4 Bloomberg School of Public Health, Johns Hopkins University, Baltimore, MD USA; 5 Department of Paediatrics & Child Health, Aga Khan University, Karachi, Pakistan; 6 Institute of Nursing & Midwifery, Dow University of Health Sciences, Karachi, Pakistan; 7 School of Population Health, University of New South Wales, Sydney, Australia; 8 Department of Pharmacy, Makerere Unniversity School of Health Sciences, Kampala, Uganda; 9 The Vaccine Confidence Project, Nairobi, Kenya

**Keywords:** Vaccination, Immunization, Vaccine acceptance, Vaccine hesitancy, Vaccine confidence, Vaccination demand, Infodemic, COVID-19

## Abstract

The first conference of the Vaccination Acceptance Research Network, *VARN2022: Shaping Global Vaccine Acceptance with Localized Knowledge*, was held virtually, from March 1^st^ to 3^rd^ 2022. This inaugural event brought together a global representation of experts to discuss key priorities and opportunities emerging across the ecosystem of vaccine acceptance and demand, from policies to programs and practice. Convened by the Sabin Vaccine Institute, VARN aims to support dialogue among multidisciplinary stakeholders to enhance the uptake of social and behavioral science-based solutions for vaccination decision-makers and implementers. The conference centered around four key themes: 1) Understanding vaccine acceptance and its drivers; 2) One size does not fit all: community- and context-specific approaches to increase vaccine acceptance and demand; 3) Fighting the infodemic and harnessing social media for good; and 4) Frameworks, data integrity and evaluation of best practices. Across the conference, presenters and participants considered the drivers of and strategies to increase vaccine acceptance and demand relating to COVID-19 vaccination and other vaccines across the life-course and across low-, middle- and high-income settings. VARN2022 provided a wealth of evidence from around the world, highlighting the need for human-centered, multi-sectoral and transdisciplinary approaches to improve vaccine acceptance and demand. This report summarizes insights from the diverse presentations and discussions held at VARN2022, which will form a roadmap for future research, policy making, and interventions to improve vaccine acceptance and demand globally.

## Introduction

Since COVID-19 was first detected in December 2019, we have seen disruption to routine immunization programs worldwide, threatening to reverse progress in the fight against vaccine-preventable diseases. The World Health Organization (WHO) and UNICEF have reported a 79% increase in worldwide measles cases in the first two months of 2022, as compared with the same timeframe in 2021 [1]. Additional fallout from the pandemic has been the proliferation of mis- and disinformation, negatively influencing confidence in vaccines [2, 3] and creating institutional distrust towards governments and health care systems [4]. The pandemic has also drawn attention to numerous barriers around vaccine access and demand, particularly in low- and middle-income countries (LMICs), which have faced highly inequitable access to and delivery of COVID-19 vaccines [5–7].

Sabin’s Vaccination Acceptance Research Network (VARN), a multidisciplinary global network of researchers, intends to provide a platform to address these issues. VARN aims to support dialogue among stakeholders from across sectors, to advance social and behavioral science-based solutions for vaccination decision-makers and implementers. In integrating global and local knowledge, VARN’s aim is to shape an interdisciplinary research agenda and provide evidence-informed recommendations to optimize policies, programs, and practice for vaccination success. Through VARN, Sabin is working to generate demand and improve vaccine acceptance and uptake globally, but with a particular focus on convening insights and resources that are rooted in on-the-ground, community perspectives towards action-oriented solutions in the often-marginalized Global South.

From March 1^st^ to 3^rd^ 2022, VARN convened an inaugural conference, *VARN2022: Shaping Global Vaccine Acceptance with Localized Knowledge* to cast a lens and generate debate and discussion on topics related to vaccine acceptance and demand. VARN2022 was a virtual conference, which brought together a global representation of experts to discuss key priorities and opportunities emerging across the ecosystem of vaccine acceptance and demand. The conference had over 750 registered attendees across 72 countries, with 306 of those logging in for sessions. Across the three days, there were 34 oral presentations, 12 poster presentations, five keynote panelists, and four technical working group discussions centered around the four key conference themes, that are discussed in detail below. Abstracts were solicited through a formal call, with submitted abstracts going through a three-phase review process including an external peer review.

The VARN2022 conference centered around four key themes. First, the critical question of “*what is vaccine acceptance??”* was considered. Understanding the continuum of vaccine acceptance and the complex, social and behavioral drivers of vaccine acceptance – or hesitancy and refusal – are vital for the implementation of impactful solutions. Second, the conference focused on the concept that “*one size does not fit all*” with regard to strategies toward increasing vaccine acceptance and demand, and the need to consider community- and context-specific approaches to build confidence in vaccines and thus improve uptake. Third, the conference discussed the current “*infodemic era*” (i.e., an overabundance of information, true, false or misleading, that complexifies health decision-making) [8] as a barrier to vaccine confidence and the specific human-centered design approaches to combat the infodemic. This theme also considered the digital tools and strategies to empower individuals and communities to counter misinformation and improve health and vaccine literacy on vaccine safety and efficacy. Fourth and last, recognizing the value of practical tools to support research and implementation initiatives, conference attendees discussed “*frameworks and best practices to ensure data integrity”* for vaccination program partners in ongoing and future activities.

The deliberations and interactions among the participants at the VARN2022 conference, though virtual, followed the standard format of presentation panels and breakout discussion sessions around the four themes. The organizers also hoped to build upon the insights shared and identify top priorities for further investment and research. This report summarizes the key learnings, by theme, from the conference around vaccination acceptance and demand which may enhance related policies, programs and practice.

## Theme 1. Understanding vaccine acceptance and its drivers

Understanding the intricacies of *vaccine acceptance* is a first step towards recognizing how it can be supported to improve vaccine uptake globally.

### Vaccine acceptance is complex and context-specific, varying across time, place, disease and type of vaccine

**Fig. 1:**
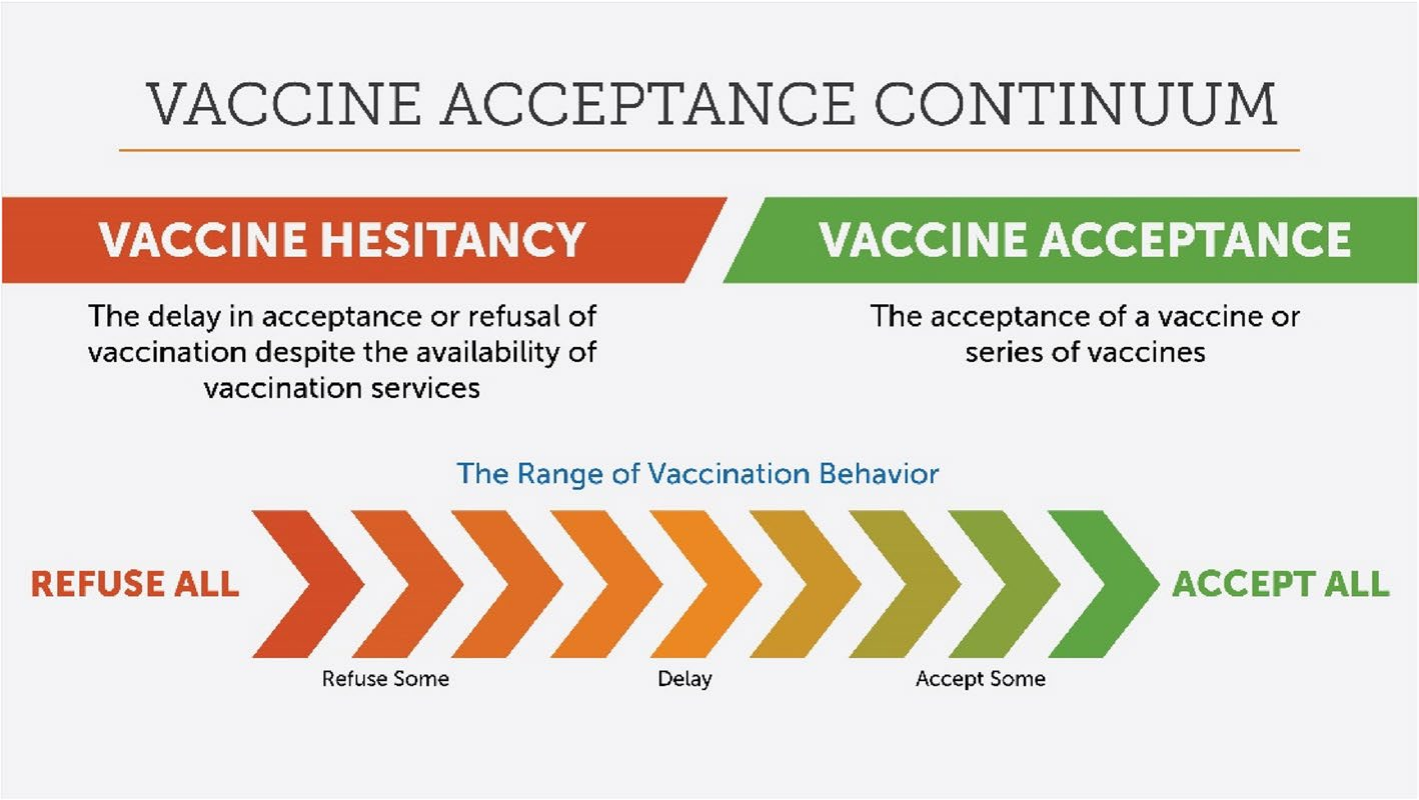
The vaccine acceptance continuum. This image was created by the Sabin Vaccine Institute. The image cites previous work that led to the development of the Vaccine Acceptance Continuum concept, including: Quinn S, Jamison A, Musa D, Hilyard K, Freimuth V. [OBK160033] Exploring the Continuum of Vaccine Hesitancy between African American and White Adults: Results of a Qualitative Study. PLOS Currents Outbreaks. 2016 Oct 31. Opel, D. J., Taylor, J. A., Mangione-Smith, R., Solomon, C., Zhao, C., Catz, S., & Martin, D. (2011). Validity and reliability of a survey to identify vaccine-hesitant parents. *Vaccine*, *29*(38), 6598–6605

Vaccine hesitancy is defined as the reluctance or refusal to accept vaccination despite the availability of vaccines [9]. Vaccine hesitancy exists on a continuum, between full acceptance and outright refusal of all vaccines. Most vaccine-hesitant people will fall somewhere in the middle of the vaccine acceptance continuum (Fig. 1).

The proportion of a population who are hard-core refusers of vaccination is an often small minority [10, 11]. For example, Dr. Ève Dubé of the Quebec National Institute of Public Health presented findings of repeated web-based surveys conducted across five waves of the COVID-19 pandemic in Quebec, Canada which found that 20% of respondents reported some vaccine hesitancy overall, while the proportion of respondents with no intention to be vaccinated remained stable at around 5%. Further, a quantitative study presented by Sunny Sharma of Ipsos Mori, UK depicting attitudinal segmentation in Kenya, Nigeria and Zambia found that only 12% of the study population was categorized as ‘strongly hesitant’, which was the smallest segment.

**Fig. 2:**
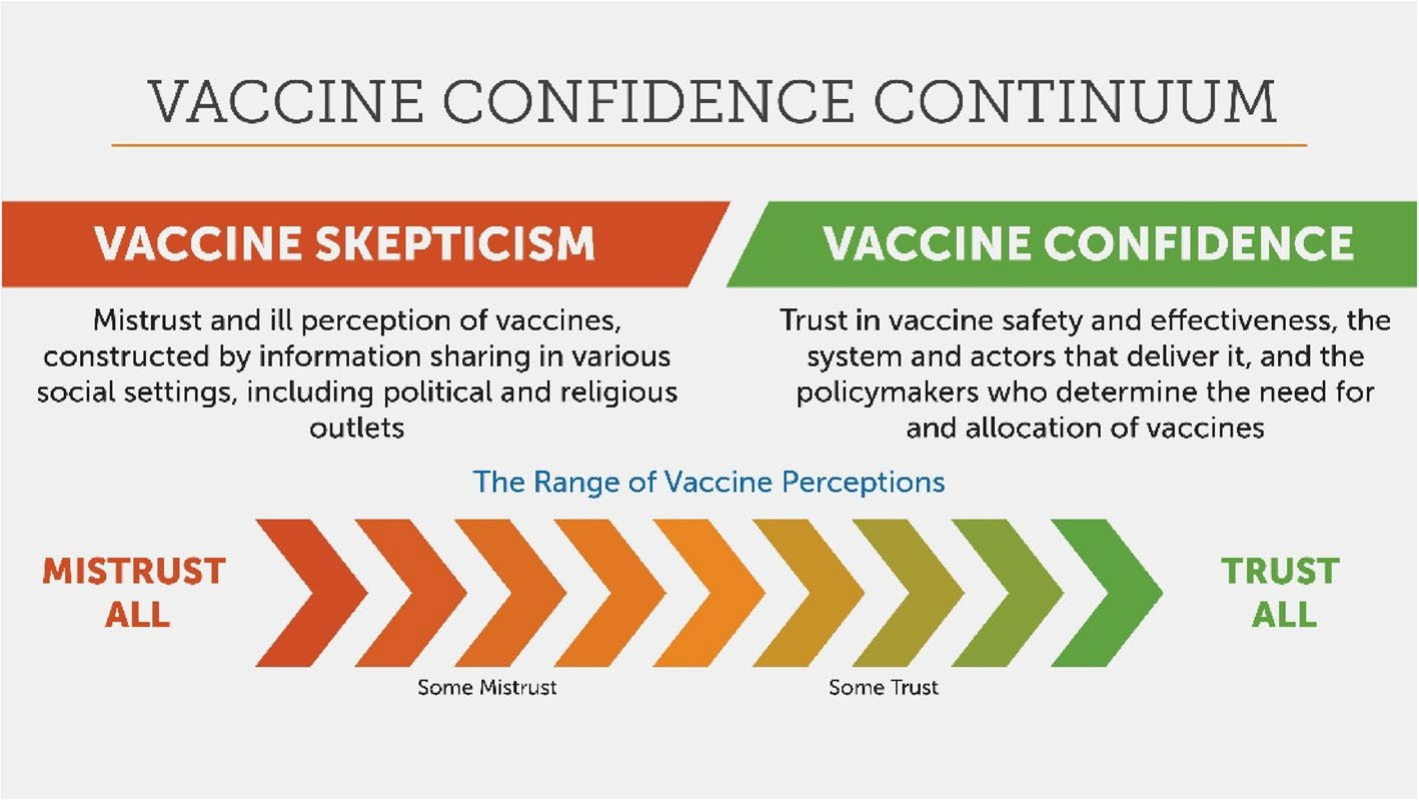
The vaccine confidence continuum. This image was created by the Sabin Vaccine Institute. The image cites previous work that led to the development of the Vaccine Acceptance Continuum concept, including: Quinn S, Jamison A, Musa D, Hilyard K, Freimuth V. [OBK160033] Exploring the Continuum of Vaccine Hesitancy between African American and White Adults: Results of a Qualitative Study. PLOS Currents Outbreaks. 2016 Oct 31

Factors influencing acceptance are complex, often specific to the context and community, and can vary across time, place, disease, and type of vaccine. For example, individuals may accept routine immunizations for their children but be hesitant to seek COVID-19 vaccination for their child or themselves [12]. During polio eradication campaigns in northern Nigeria and northern India, communities were refusing polio vaccine while at the same time demanding measles vaccine [13]. Given this complexity, Sabin and key global stakeholders use the term *vaccine acceptance* rather than *vaccine hesitancy,* which acknowledges this complexity and the broader structural factors that may influence an individual’s decision to vaccinate or ability to become vaccinated. Vaccine confidence is particularly influential on vaccine acceptance and is one of the “5Cs” of psychological antecedents to vaccination [14]. Like vaccine acceptance, vaccine confidence lies on a continuum and may be gained and lost over time (see Fig. 2).

Philip Wollburg (Data Production and Methods Unit, World Bank, USA) shared findings from longitudinal surveys conducted across Burkina Faso, Ethiopia, Malawi, Mali, Nigeria, and Uganda by the World Bank, which highlighted and elucidated key patterns behind low COVID-19 vaccine acceptance and confidence. While sociodemographic patterns of vaccine acceptance varied across countries, there was typically higher hesitancy in urban areas and among populations with higher levels of education and those with greater household incomes [15]. Vaccine acceptance was generally high, with 80% of surveyed people willing to be vaccinated in all countries except Mali, but there were notable variations amongst countries and over time [15]. In Ethiopia, Nigeria and Uganda attitudes towards COVID-19 vaccination remained stable, while in Burkina Faso and Malawi switching of attitudes was more evident. Common reasons for low COVID-19 vaccine acceptance across the surveyed countries related to concerns about vaccine safety and potential side effects [15].

The COVID-19 pandemic has also had an impact on vaccine acceptance more generally with previous notions of vaccine hesitancy seen primarily at an individual level, often driven by influential individuals, such as faith leaders with negative opinions of vaccines [16]. However the pandemic, and the accompanying infodemic, has sparked greater polarization of opinions based on ideological differences, with pandemic denial even propagated by political leaders [17, 18]. Yet despite this, COVID-19 has led to some positive changes in attitudes to vaccination, bringing awareness to and opportunities for engagement on issues around vaccination, including the establishment of initiatives to improve vaccine equity [19, 20]. At VARN2022, discussions of vaccine equity brought forth the concept of the “intention-action gap” – wherein an individual’s intention to vaccinate does not automatically translate into becoming vaccinated, such as when an individual may face difficulties in getting to a vaccination center, despite wanting to get vaccinated. This raises the issue of vaccine access and demand and its relationship to vaccine (in)equity and acceptance, a concept that is further discussed in the next section.

### Vaccine inequity and weakened health systems are barriers to vaccination access and demand

The challenges of vaccine inequity and barriers to access negatively impact vaccine acceptance and demand. Many communities face systemic barriers when accessing healthcare and vaccination – particularly marginalized groups, including some people from ethnic minority communities, Indigenous communities and refugees. Central to VARN2022 was sharing insights on these barriers garnered from vaccination research and program implementing partners working closely with marginalized communities, often located within LMICs.

It is important to recognize that willingness to vaccinate is often high in LMICs. A recent survey of 10 LMICs reported that average willingness to take a COVID-19 vaccine was 80.3%, compared with 64.6% and 30.4% across populations surveyed in the United States and Russia, respectively [21]. However, willingness to vaccinate cannot translate into vaccination uptake without strong health systems and sufficient resources in place to coordinate and enable widespread vaccination program rollout.

During the COVID-19 pandemic, many LMICs have faced systemic issues that have hindered vaccination access and rollout. VARN2022 keynote speaker, Robert Kanwagi (Consultant for Gavi, the Vaccine Alliance) shared that while the Democratic Republic of the Congo received 6.2 million doses of COVID-19 vaccines in 2021, only 2.2 million doses were utilized due to practical issues with vaccine rollout, despite high willingness for vaccination across the population. In a presentation on the last-mile delivery of COVID-19 vaccines in Sierra Leone, Dr. Ahmed Mushfiq Mobarak (Yale University School of Management, USA) described how it can take an average of three hours each way to reach a local vaccination center in rural areas of Sierra Leone. As such, an overnight trip costing US$ 6.50, in a region where people live on US$ 1 a day, is not feasible or affordable for people living in these areas, despite willingness to get vaccinated.

It is therefore important to recognize the difference between true vaccine hesitancy and low vaccine demand and uptake that is due to systemic barriers to vaccination and resource constraints. Solutions must avoid placing blame on individuals without recognizing the wider interplay between the individual and broader health system and environment.

## Theme 2. One size does not fit all: community- and context-specific approaches to increase vaccine acceptance and demand

A central theme to VARN2022 was the concept that there is no “*one size fits all*” approach to understanding or improving vaccine acceptance and demand around the world. Communities have diverse and unique characteristics, which require tailored solutions to address the specific barriers faced. As a result, context-specific, community-centered strategies are essential to increasing vaccine uptake and sustaining demand. The conference provided a forum for presenters to share learnings from diverse settings around localized solutions to increase vaccine acceptance and demand, particularly in low-resource settings.

### Community-centric methods are essential to increase vaccine acceptance and demand, particularly among marginalized and hard-to-reach populations

**Fig. 3:**
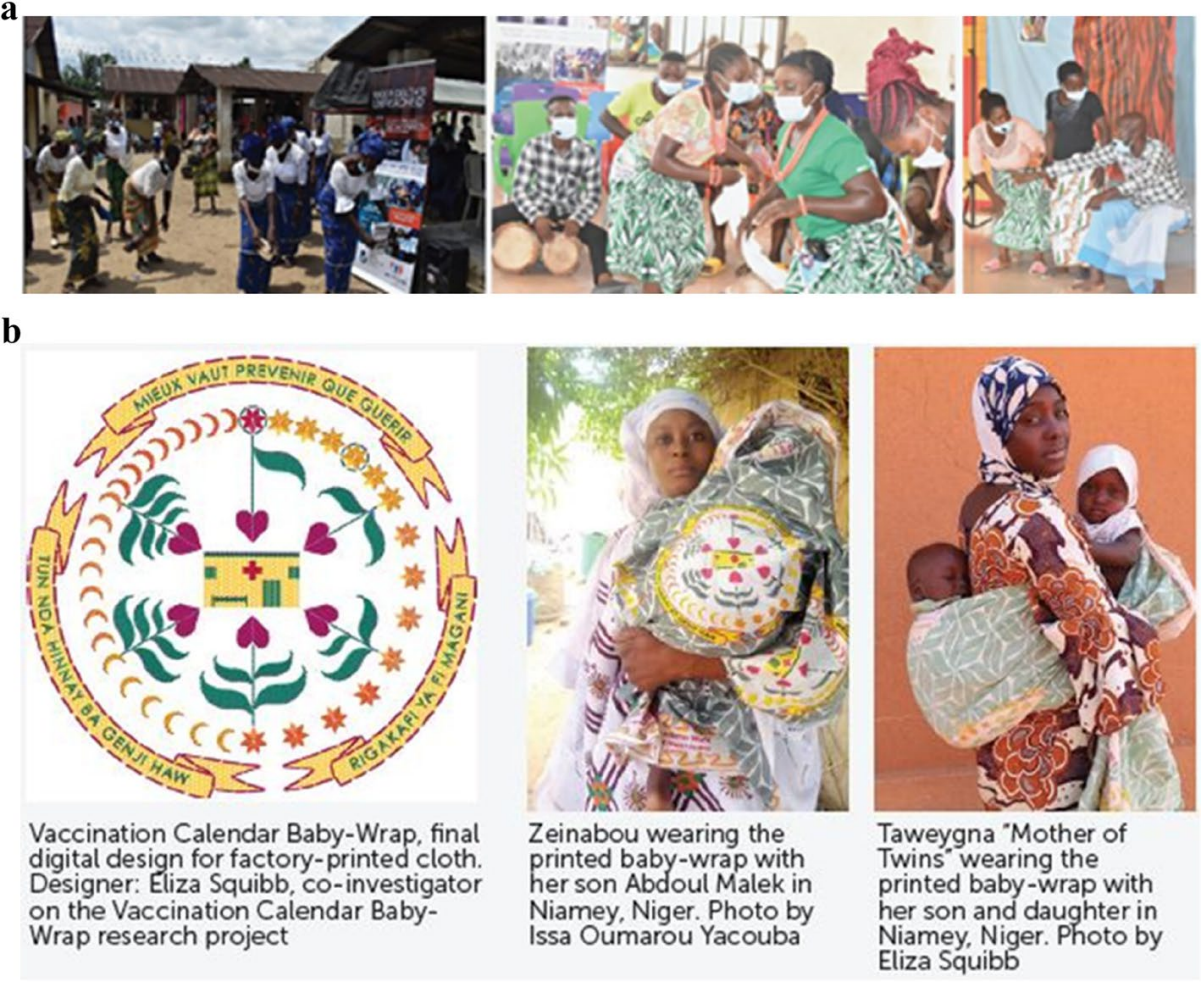
Community-centric approaches to increase vaccine acceptance and uptake. **a** Community theater performances in Nigeria as part of a community theater for immunization (CT4I) intervention. Explicit written permission was obtained from Chijioke Kaduru, Corona Management Systems, Nigeria to publish these photographs in an open-access journal. **b** The “Vaccination Calendar Baby-Wrap” initiative to improve vaccination coverage in Niger, West Africa. Explicit written permission was obtained from Eliza C. Squibb, Massachusetts Institute of Technology, to publish these photographs in an open-access journal

Central to understanding the root causes of low vaccination within communities is working directly with the community itself, to fully understand the sociocultural factors influencing vaccine acceptance and demand. Presenters shared learnings from work with diverse communities, including populations within urban, peri-urban, and rural settings; marginalized and displaced groups; and “zero-dose” children who have not received any routine immunizations. Three examples of successful community-centric approaches to increase vaccine acceptance and uptake were presented at the conference. Nadine Skinner, (Stanford Center for Health Education) and Anne Kraemer Diaz (Maya Health Alliance/Wuqu’ Kawoq) presented on work co-designing messaging with Indigenous Maya in the Central Highlands of Guatemala. A lack of health information in native languages is a particular barrier for the Indigenous Maya population in Guatemala. To address this large unmet need, a project was undertaken to improve local health messaging through co-designing culturally relevant images and messages in local Mayan languages with community leaders. These culturally relevant and appropriate for low literacy populations messages in local languages were disseminated over social media [22]. Additional presentations on this theme included a community theater intervention in the Niger Delta region of Nigeria by Dr. Chijioke Kaduru (Corona Management Systems, Nigeria) and a project by Eliza C. Squibb (Massachusetts Institute of Technology, USA) and Mika Kondo Kunieda (Keio University, Japan) in which a vaccination calendar baby wrap was co-designed with local artists, mothers, and healthcare workers in Niger. More details about these presentations are shown in Fig. 3.

When working with communities to improve vaccine acceptance and demand, presenters highlighted the importance of developing pragmatic, user-friendly and contextually relevant interventions that are acceptable to the community. Simplified interventions avoid burdening the community and increase the likelihood of implementation and sustained change. To ensure interventions are long-lasting and vaccine knowledge stays within the community, strategies also need to be adaptable to accommodate contextual shifts that the community may encounter or experience. Empowering local community leaders and stakeholders through capacity building efforts is therefore essential to provide the community with the tools and knowledge to continue projects once original (often external) partners disengage. These steps are also essential for the decolonization of research and programs.

Health and vaccine-related messaging also need to be delivered by trusted individuals in the community, and VARN2022 presenters across global regions spoke about the need to identify and engage with key community influencers who already have strong relationships with their community.

### Healthcare workers are trusted messengers often able to improve attitudes and intentions around vaccination, but they also need training and resources

Healthcare workers, particularly community health workers, are generally seen as trusted messengers who have the power to improve attitudes and perceptions around vaccination. However, healthcare workers can also be recipients of and influenced by misinformation (incorrect or misleading information that is not intentionally spread) and disinformation (deliberately deceptive information). Training healthcare workers on vaccine literacy and best practices for countering mis- and disinformation is generally not routine practice, which can lead to community vulnerability to misinformation. This vulnerability is particularly present for informal healthcare workers or traditional healers, who in some communities are more trusted than formally trained and certified health practitioners.

Healthcare workers who are misinformed or hold vaccine-hesitant views, including those who refuse vaccines, can prove detrimental to vaccination campaigns, and potentially initiate a negative shift in vaccination attitudes among their colleagues, patients, and community. At VARN2022, Dr. Benson Wamalwa and Dr. Caroline Aura (University of Nairobi, Kenya) shared research reporting that vaccine acceptance was only 26% among community health workers in Trans-Nzoia County, Kenya – substantially lower than among the general population. As a result, there is a particularly urgent need to better train healthcare workers on health and vaccine literacy, to ensure that healthcare workers serve as a trustworthy knowledge base for their community, particularly around vaccine eligibility and safety.

### Non-traditional actors also have enormous power to address and improve vaccine acceptance and demand in the community

The importance of working with respected and trusted community members outside of the formal health system was repeatedly discussed across the conference. Deeply rooted community mistrust in governments and health systems often makes non-traditional actors more trusted – and effective – communicators. As a result, working with community-level change-makers, such as faith leaders, schoolteachers, civil society activists, traditional healers, and even youth, should be a key component of communication and education initiatives. Empowering such individuals with knowledge on vaccine benefits can help create strong vaccine champions with widespread community reach. As shared by Ashima Gulati (#HumHongeKaamayaab, India) in a moderated discussion at VARN2022, a single speech on vaccination from a religious leader in India led to a positive attitudinal shift, with 95% of vaccine-hesitant individuals subsequently willing to accept a COVID-19 vaccination.

### Social and gender dynamics can act as barriers to vaccine uptake in women, which should be addressed through tailored interventions

Several presentations at VARN2022 discussed how social norms and gender inequity create additional barriers around vaccine access for women and potentially for their children. Although not geographically exhaustive, presenters shared case studies that found lower levels of COVID-19 vaccine acceptance in women compared to men in countries across sub-Saharan Africa and India, for diverse reasons including lack of information, fear of negative effect on reproductive health and fertility and limited autonomy to seek information and vaccination services [23].

These identified concerns around reproductive health and fertility indicate that health workers may also require additional training to ensure they have the necessary tools and information to address these specific concerns, particularly vaccine eligibility and safety during pregnancy and breastfeeding. Interventions must consider local values, traditions, and cultural practices, to make sure solutions that empower women are feasible and well received by the community.

Demonstrating the impact of community-led interventions, Rubina Qasim (Institute of Nursing & Midwifery, Dow University of Health Sciences, Pakistan) shared the positive impact of a project that worked with the local community to co-design and test social and behavioral interventions to counter COVID-19 related misinformation in Landhi town, Karachi, Pakistan. A female study participant shared, “*In our society, females are given less priority in decisions. The thing we like about this study was our active involvement from day one and respecting our culture, autonomy and giving us opportunity to decide and act along with other stakeholders and researchers. This was the reason why this approach is greatly needed in the community*”.

Vaccination demand creation interventions may also strategically target men and fathers, as men often hold the power to enable or block access to vaccination for the household. A poster presentation at VARN2022 by James Bell et al. (Ipsos, UK) shared findings from a study conducted in Nigeria, Uganda and Guinea on the role of fathers in the uptake of early childhood immunizations. Evidence-based recommendations included linking vaccination with the health and financial success of the family and ensuring that vaccine information is disseminated by trusted, community influencers to encourage fathers to allow the completion of their children’s immunization schedules.

### Communities of practice are an essential tool to drive community participation in interventions around vaccine acceptance and demand

Communities of practice are groups of people that come together to fulfil a shared purpose around a community issue or interest and share information and expertise [24]. Such groups can facilitate peer-to-peer learning between members. Communities of practice were first used to describe “learning communities” in the workplace, but the concept has also been applied to facilitate learning among healthcare workers and to address health issues in the community [25–28].

Communities of practice are an important tool for elevating community concerns and knowledge from the ground up to policy and program decision-makers and keeping those parties accountable. As such, they play a key role in enabling community-centric approaches in program design, translating knowledge into action and facilitating a bottom-up and top-down feedback loop.

The role of communities of practice in vaccine acceptance and demand was explored in a breakout discussion session at VARN2022. Breakout group participants discussed the existing landscape of communities of practice and networks focused on vaccination and best practices to implement an informative, dynamic, and needs-based cycle of change around vaccination. Global communities of practice, such as Sabin’s Boost community [29], were highlighted as important to facilitate global knowledge sharing and collaboration, and understanding of different cultures, contexts and languages.

## Theme 3. Fighting the infodemic & harnessing social media for good

The current “infodemic” era means that public health issues are accompanied by an overabundance of information, much of which can be false or misleading, particularly on social media [30, 31]. As stated by WHO Director-General, Dr. Tedros Adhanom Ghebreyesus, *we are not just fighting an epidemic – we’re fighting an infodemic* [32]. Improving vaccine acceptance and demand therefore requires solutions that address the problems created by the infodemic directly. As such, another major theme of the conference looked at tools to fight the infodemic and how to build capacity for online users to identify misinformation. Participants specifically considered how social media can be harnessed to share accurate information about the COVID-19 pandemic and COVID-19 vaccination, including messaging to promote vaccine acceptance and demand.

### Misinformation flourishes in the absence of quality information, particularly on social media, and agile research practices are needed to fill information voids

High levels of vaccine-related misinformation are circulating globally, disproportionately affecting marginalized populations. These populations have often been neglected or mistreated by government health systems, resulting in less trust in and familiarity among such groups with official health information sources.

Speakers at VARN2022 working with communities in Pakistan, Guatemala and Uganda reported high levels of misinformation around vaccines [33]. Findings around common myths and misinformation related to COVID-19 and the COVID-19 vaccines were highlighted in these presentations and spanned safety issues (e.g., *vaccines cause infertility in one or both sexes, vaccines are unsafe, cause deaths*), government conspiracies (*COVID-19 is a government hoax, tracking microchips are implanted during vaccination*) and religious concerns (*vaccines are against the will of God/cause you to become marked by the devil*).

Structural issues in research and policy were also highlighted as contributing to the infodemic. The lengthy process for ethical approvals and implementation of research protocols and subsequent publication of research findings can create information voids. Gaps in reliable information provide space for “bad actors” to fill with incorrect or misleading information [34]. For example, a “wellness” influencer may create social media posts containing false information about the dangers of COVID-19 vaccination to promote their own alternative health products. Therefore, there is a need to balance scientific rigor with expedited outputs to ensure scientific findings, and the subsequent public health guidance, are available to the public in accessible and timely formats – including utilizing novel media forms like social media.

### There is a need to build capacity and train “infodemiologists” to identify and combat misinformation

Central to tackling the infodemic is building people’s ability to spot and combat misinformation when encountered in all its various forms. The conference looked at practical tools to build capacity, through both the dissemination of tools to combat misinformation and training “infodemiologists” to recognize and combat misinformation.

Infodemiologists act as liaisons between different stakeholders in public health communication and the community to impact the overall information environment. In online settings, infodemiologists can take the role of moderators and help reframe discussions to promote engagement and understanding instead of divisiveness. This cadre of workers can also provide empathetic, non-judgmental listening to community issues and help communicate those concerns back to public health decision-makers. As such, a range of stakeholders have skills suited to be infodemiologists – health workers, community leaders, field epidemiologists – but most importantly, they need to be trusted by the community, have strong communication skills and be willing to act as liaisons.

At VARN2022, Tina Purnat (WHO Infodemic Management team in the Unit for High Impact Events Preparedness) shared that WHO has a dedicated infodemic management team, which has been working with key stakeholders to develop an infodemic management framework [35]. As part of this initiative, WHO has developed tools to train and support field infodemiologists and is working to create evidence-informed policies and systems to prevent harm from current and future infodemics. An infodemic management training toolbox is readily available to support capacity building, health system strengthening and developing communities of practice. The toolbox includes a self-paced, open online training “Infodemic Management 101” as well as offline, “train the trainers” workshops and technical capacity building at the country level [36]. Dr. Abdul M. Kazi (Aga Khan University, Pakistan) also spoke about the need for investment in tailored training for local doctors and healthcare practitioners using digital health tools and interventions on how to create vaccine awareness and advocacy within rural and peri-urban settings and how to increase literacy regarding the quality, safety, and efficacy of vaccination.

### Social media and digital health platforms can be leveraged to combat the infodemic through approaches that have the right appeal, message and messenger

Given the context-specific nature of vaccination acceptance and demand, public health messaging must be persuasive and tailored to resonate with the target population. A successful communication strategy for parents in rural Pakistan will likely require different content and methods compared with a successful campaign to reach teenagers in the USA. It is important to consider how a message is framed, in terms of the informational content (i.e., main takeaway point – “get vaccinated to protect against COVID-19”), and appeal (i.e., the form of messaging that garners the most attention – such as appealing to a person’s morality, emotions, rationality, etc.) [37]. Other critical elements for health messaging are the right messenger for the setting (i.e., the most influential type of person disseminating the information) and best method of delivery (e.g., printed infographic vs social media).

Research presented by Dr. Dena Gromet (University of Pennsylvania) and Dr. Daniel Erchick (Johns Hopkins University) discussed specific approaches that have shown promise. The importance of empathy was highlighted across the conference as critical to connect with people and build trust so that health messages resonate. Other approaches that were noted as impactful were SMS-based “nudges” that indicate a vaccine has been reserved for an individual and using messages that are “health-outcome” focused. Social media should also be viewed as a powerful tool to help combat the infodemic, given the popularity and reach of platforms like Facebook, YouTube and Twitter [37].

Social listening, the process of identifying what is being discussed within a community, was also highlighted as an important tool to understand people’s vaccination-related concerns, questions and information gaps that need addressing in a timely manner. Recognizing the value of social listening for the rapid understanding of public health issues, WHO has harnessed artificial intelligence technology to develop a platform for online social listening around COVID-19. The platform, known as Early AI-supported Response with Social Listening (EARS), summarizes real-time information about COVID-19 conversations in public online spaces to help inform the infodemic response [38]. However, presenters at VARN2022 also discussed the need for an ethical framework around social listening and subsequent targeted interventions, to avoid the perception of unethical influence from public health organizations in online spaces.

**Fig. 4:**
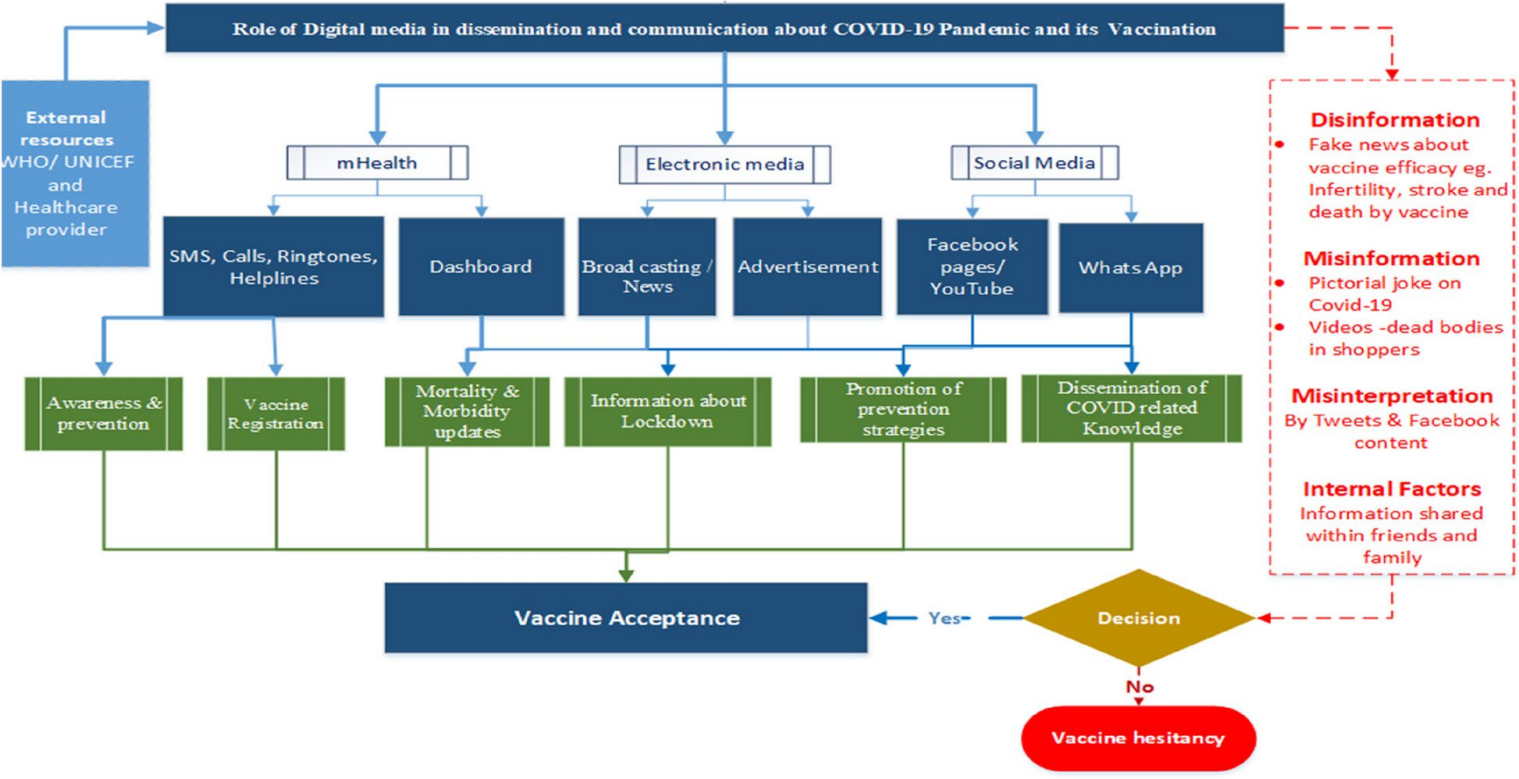
Pathway for digital information and dissemination of COVID-19-related communications. Dr. Abdul M Kazi is a co-author on this paper and has given approval of the content of this manuscript for an open access publication, including this figure, *in BMC Proceedings*

Dr. Abdul M. Kazi further recommended designing low-cost digital health solutions to identify and overcome mis- and disinformation barriers related to the COVID-19 pandemic and vaccination. These included mobile phone-based caller tunes, text and automated call-based messages serving as behavior change applications. At VARN2022, Dr. Kazi presented a pathway for digital information and communications to be disseminated through these interventions (shown in Fig. 4).

### Training and science-based gaming can be effective tools to fight the infodemic

Numerous applications, courses and games have been developed to fight the infodemic. These tools help educate users on how to identify misinformation, provide tools to develop interpersonal communication skills to effectively support conversations around COVID-19 vaccination, and provide tips and training to combat misinformation in real-life. For example, two short games – Bad News and GO VIRAL! – have been developed by the Social Decision-Making Laboratory at the University of Cambridge to help players identify misinformation using social psychology [39, 40].

**Fig. 5:**
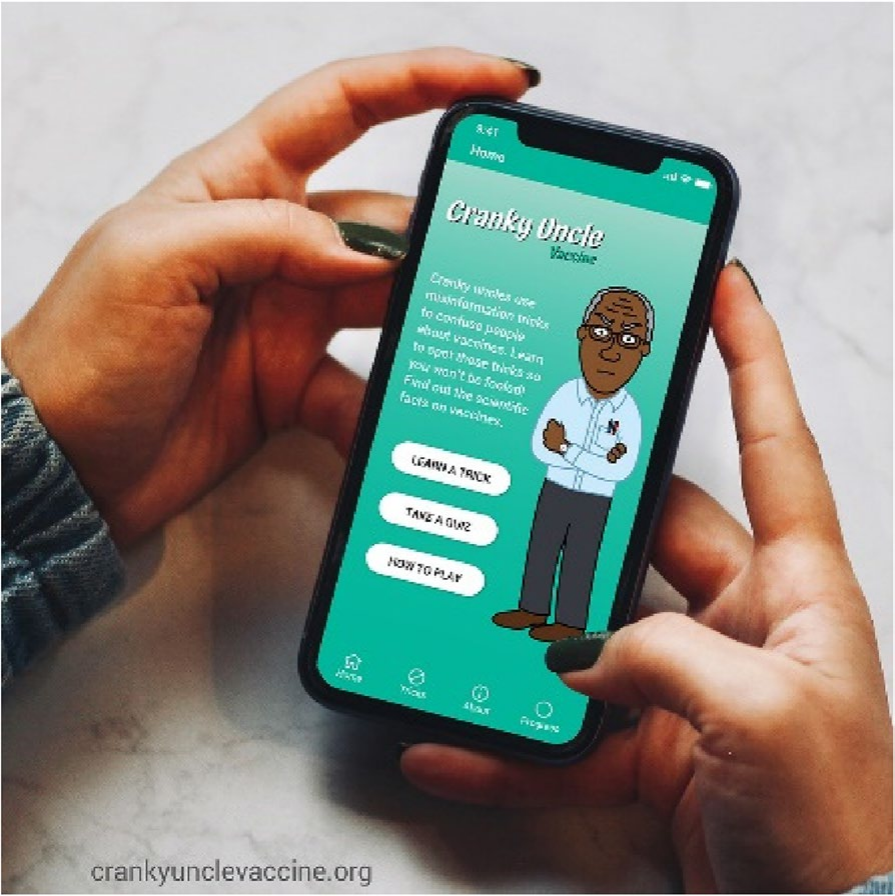
Snapshot of the Cranky Uncle game. Explicit written permission was obtained from Dr. John Cook to publish these snapshots of Cranky Uncle in an open access publication. Credit should go to Wendy Cook for creating the photo

One of the tools discussed at VARN2022 included an artificial intelligence chatbot named “VIRA”, developed by Johns Hopkins University to provide trustworthy information on COVID-19 vaccines [41]. The chatbot is adaptive and listens to users’ questions and provides immediate answers. Short online games are another approach that show promise by providing a fun, accessible way for people to practice critical thinking skills. One of these online games, “Cranky Uncle”, was discussed by its creator, Dr. John Cook (Monash University Climate Change Communication Research Hub, Australia). Cranky Uncle is a free game built for smartphones, which aims to combat misinformation using *inoculation theory* [42]. This theory, when applied to misinformation, refers to the concept that exposing people to a weakened form of misinformation builds up their “mental antibodies”, providing immunity to misinformation over time, so they are more able to identify misinformation when encountered in the real world, and less likely to be misled [43]. The game uses a “*cranky uncle*” cartoon figure who mentors players throughout the game and explains techniques used by science denialists, using humorous examples in analogous situations (Fig. 5). A vaccine module of the *Cranky Uncle* game is currently in development and will be pilot tested in various global regions with translated scripts.

In addition, Johns Hopkins University has developed accessible, free online resources for frontline health workers and the general population to help address misinformation. There is a course available providing coaching on how an individual may talk to peers about vaccines, focusing on using empathy to build trust [44], and an online training for frontline health workers to assist them with identifying and responding to misinformation in their communities [45].

## Theme 4. Frameworks, data integrity & evaluation of best practices

The final theme of the conference considered evidence-based tools, frameworks and best practices to increase vaccine confidence and acceptance.

### There are key social and behavioral drivers of vaccine uptake that can be targeted in communications for change

**Fig. 6:**
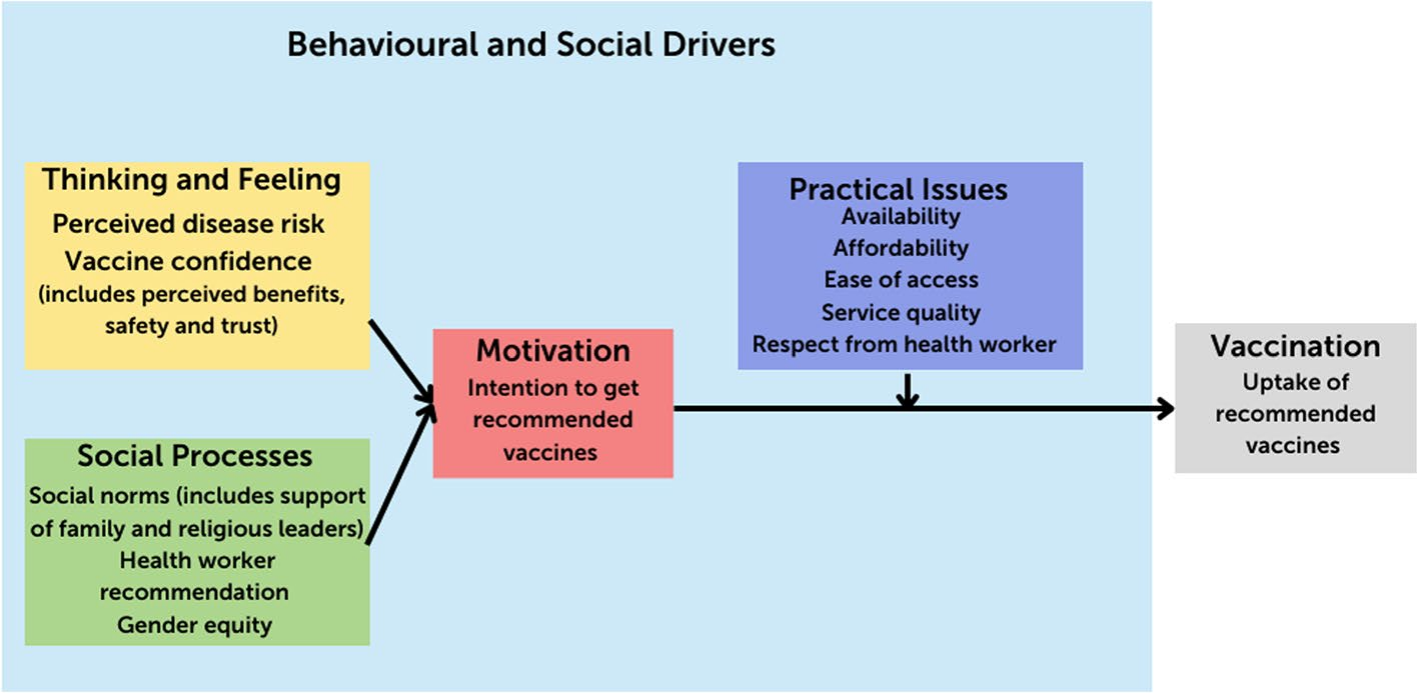
The WHO Behavioural and Social Drivers (BeSD) of Vaccination Framework. The publication from which this figure originates comes from the WHO publication, *Behavioural and social drivers of vaccination: Tools and practical guidance for achieving high uptake*, found at https://apps.who.int/iris/bitstream/handle/10665/354459/9789240049680-eng.pdf?sequence=1&isAllowed=y. This publication is open and freely available on the WHO website. We have added a citation that includes the link to this publication directly under this figure. We also received verbal permission from Lisa Menning of WHO

Effective frameworks can be useful to understand drivers of vaccine acceptance and demand in different populations and to develop appropriate solutions. WHO has undertaken work to understand the behavioral and social drivers of vaccine uptake and develop tools to support programs in measuring and addressing these factors. As shared at the conference by Lisa Menning (WHO Headquarters Department of Immunization, Vaccines, and Biologicals, Switzerland), WHO developed an evidence-based framework to measure and address hesitancy in ways that can be tailored to the local setting [46]. The WHO framework of Behavioural and Social Drivers (BeSD) of Vaccination constitutes four key domains to evaluate and understand factors contributing to under-vaccination (depicted in Fig. 6) [46].

The WHO working group has also developed and validated a set of tools – available on the WHO website – to support programs and partners in measuring and addressing reasons for vaccine hesitancy and the systemic monitoring and evaluation of data over time [46]. The set of tools includes quantitative surveys, qualitative interview guides, and implementation guidance to support partners with planning data collection, data analysis, and integration into program activities.

### Best practices need to be established for data collection, reporting and database harmonization

Understanding barriers to and drivers of vaccine acceptance and demand in a community requires the collection of quality data on vaccination access and immunization coverage rates, as well as standardized methods for measuring personal beliefs, perceptions, and cultural sentiments. However, challenges around how data are collected, reported and harmonized can compromise understanding of and solutions for low rates of vaccine acceptance. As a result, there is a need to improve data quality and confidence in the data collection process, with consideration of how different protocols may lead to under or overreporting of outcomes and/or introduce biases. For example, collecting data through telephone surveys may lead to oversampling of certain parts of the population (e.g., those most likely to be at home to answer a telephone survey), potentially affecting the generalizability of findings. Similarly, social and cultural norms may affect how truthful people are when asked about vaccination.

Furthermore, building data at the national level can mask community-level nuance, highlighting the importance of data collection at the local level, using tools tailored for specific settings. For example, developing surveys specifically targeted to women will require consideration of how best to reach them within the community. Pooling and/or layering different datasets, including topographical and economic information, can also create a richer picture of the barriers and enablers of vaccine acceptance and uptake within communities. However, careful consideration of privacy and confidentiality is essential when using large and diverse datasets to understand low vaccine acceptance, particularly within marginalized communities.

There was agreement that the lack of database harmonization is a particular challenge for vaccination programs in LMICs. One example shared at the conference around COVID-19 vaccine rollout in South Africa highlighted the difficulties that can arise from segmented databases. In this case, the COVID-19 vaccination program was initiated with only one approved brand of vaccine, with additional brands becoming available at later phases of implementation. However, as vaccination records were held separately by each vaccine manufacturer, it was not possible to track the proportion of the population who received boosters of a different brand to their primary vaccination series (heterologous boosters).

Global alliances like VARN, which bring together key players in the field, therefore have a central role to play in supporting best practices and pooling of resources, for the greatest impact on vaccine acceptance and demand. WHO is also developing a number of tools to help facilitate better data collection, for example, templates to convert findings into charts and briefing notes on evidence-based interventions that have been shown to increase vaccine uptake [46].

### Transdisciplinary and multi-sectoral collaboration needs to be leveraged to boost vaccine awareness and uptake

Across the conference, there was agreement that tackling low vaccine acceptance and the infodemic requires a multi-disciplinary and multi-sectoral collaborative approach, as the associated drivers are multifactorial and varied. As such, we need to leverage multi-level stakeholder skillsets from disciplines as diverse as public health, the social and behavioral sciences, journalism, and data/computer science. Approaches also need to be inclusive of academia, civil society, the public and private sectors, among others.

With this aim in mind, VARN2022 provided a forum for presenters to share lessons learned from other health challenges and disciplines that could be applied to vaccine hesitancy. Dr. Janan Dietrich (Bio-Behavioural Research Division Perinatal HIV Research Unit, South Africa) discussed how learnings from HIV candidate vaccine acceptance could be applied to COVID-19 vaccine acceptance among youth in South Africa [47]. Dr. Susanne B Montgomery (Loma Linda University School of Behavioral Health, USA) also shared how the community resiliency model, a set of easily learned self-care skills, can be translated to combat low levels of vaccine acceptance [48].

Insights were also shared around how private sector techniques, long used to understand and micro-target customers, can be leveraged for public health initiatives. Sunny Sharma (Ipsos, UK) discussed how attitudinal segmentation, a practice borrowed from the commercial sector which clusters people together with similar attitudes and behaviors, can be used to develop tailored, impactful messaging around vaccination. Kenneth Davis (Fraym, USA) discussed the application of geospatial mapping and data analytics to support public health stakeholders s in tailoring mass vaccination campaigns to key population segments by providing granular, community-level data, for example, on the determinants of vaccine hesitancy.

## Conclusions

**Fig. 7:**
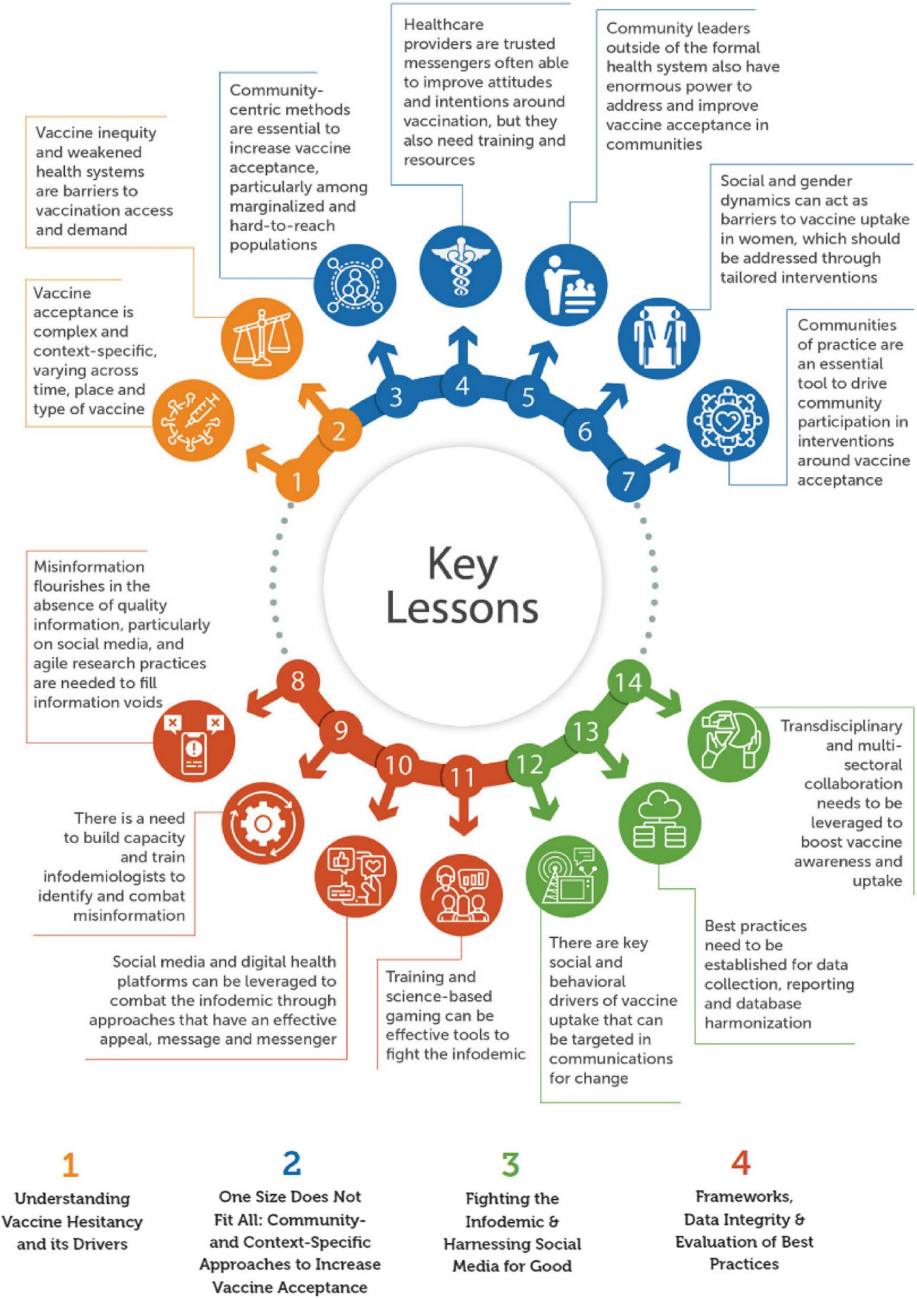
Key learnings from VARN2022 by theme. This image was conceptualized and created by the co-authors from the Sabin Vaccine Institute

The inaugural VARN conference *VARN2022: Shaping Global Vaccine Acceptance with Localized Knowledge* provided a wealth of insights around the current landscape of vaccine acceptance and demand, on a global and local scale. Across the conference, presenters considered the drivers of and strategies to increase vaccine acceptance and demand relating to COVID-19 vaccination and other life-course and child routine immunizations. Figure 7 highlights the key lessons shared through the conference. Altogether, VARN2022 highlighted the need for a human-centered approach to address low levels of vaccine acceptance, recognizing that tailored, people-focused and community-specific solutions are needed to drive confidence in vaccines.

It was also clear that engaging multi-sectoral and transdisciplinary perspectives, tools and systems will be critical to the success of work to improve vaccine acceptance. VARN2022 strived to initiate such collaborations to bring about meaningful changes and create a platform for continued engagement and inquiry into vaccination practices and policy. The insights gathered at VARN2022 have set the stage for the development of a roadmap for research, policy making, and program decision-making in the future.

## Data Availability

As a report article on conference proceedings, all information – including recordings of presentations – can be found at VARN 2022—Vaccination Acceptance Research Network (vaccineacceptance.org).
